# The geriatric nutritional risk index mediated the relationship between serum uric acid and hypertension: a mediation analysis

**DOI:** 10.1186/s12877-021-02483-5

**Published:** 2021-10-02

**Authors:** Zhongnan Cao, Sui Dai, Xun Liu

**Affiliations:** 1 Department of Cardiology, Tianjin Fifth Central Hospital, 300450 Tianjin, China; 2 Department of Laboratory, Tianjin Fifth Central Hospital, 300450 Tianjin, China; 3 Department of Ultrasonics, Tianjin Fifth Central Hospital, No 41 Zhejiang Road, 300450 Tianjin, China

**Keywords:** Geriatric Nutritional Risk Index, serum uric acid, hypertension, mediation effect

## Abstract

**Background:**

The elevated serum uric acid (SUA) is associated with an increased risk of hypertension and nutritional status. Malnutrition might modify the association of SUA with hypertension. Therefore, the aims of this study were to examine the mediation effect of malnutrition on the association of SUA with the risk of hypertension in Chinese population.

**Methods:**

The study was based on the China Health and Nutrition Survey in 2009. Participants aged ≥ 60 years with complete analyzed data were eligible. The Geriatric Nutritional Risk Index (GNRI) was calculated by serum albumin (ALB) and BMI. Participants were identified as hypertension if systolic blood pressure ≥ 140 mmHg and/or diastolic blood pressure ≥ 90 mmHg or receiving antihypertensive drug.

**Results:**

There were 2371 participants included in the final analysis. In total, there was a significant mediation effect of the GNRI on the relationship between SUA level with hypertension (*P* < 0.001; *OR*: 1.096; and *95 % CI*: 1.048–1.146). And the proportion mediated was 17.77 %. The results stratified by sex were consistent with those of total population. The significant mediation effects of the GNRI were found in the 60–69 years and 70–79 years groups (*P* = 0.002 and 0.032; *OR*: 1.099 and 1.075; and *95 % CI*: 1.036–1.165 and 1.006–1.148, respectively) but not in the 80–99 years group (*P* = 0.303). The proportions mediated were16.22 % and 18.36 %, respectively.

**Conclusions:**

The GNRI can mediate and account for approximately 17.77 % of the relationship between SUA level and the risk of hypertension. And this mediation effect was fully observed in both males and females, especially in the 60–79 years population.

## Background

Hyperuricemia was prevalent worldwide and has adverse effect on the developments of hypertension, diabetes, and dyslipidemia.[1] Therefore, it has been a major public health issue. There were many studies to report that elevated serum uric acid (SUA) level contributed to the increased incidence of hypertension and cardiovascular mortality.[2–6] Although the association of elevated SUA level with incident hypertension was established, it is suggested that several metabolic factors may mediate the pathways from SUA to hypertension.[7] Previous study declared that hyperinsulinemia is associated with the reabsorption of SUA in the obese subjects. [8] Furthermore, fat cells might modify the association of hyperuricemia with hypertension.[8, 9].

Previous studies reported that elevated SUA level was associated with nutritional status.[10, 11] Since consumption of purine-rich meat, seafood, and fat could increase SUA level, a low SUA level implied inadequate protein and calorie intake.[12] Therefore, it was likely that protein-energy wasting might cause a low SUA level in the malnourished participants. Furthermore, in a recent study, there was a lower SUA level in the malnourished population than the healthy population.[13] On the other hand, nutritional status was identified as an independent predictor of hypertension.[11, 14] Therefore, there might be interplay between SUA level and nutritional status on hypertension.

In view of the mentioned above, we hypothesized that nutritional status might mediate the relationship between SUA level and the risk of hypertension. However, until now, there was no study to investigate the mediation effect of malnutrition on the relationship between SUA level and the development of hypertension. Since the Geriatric Nutritional Risk Index (GNRI) was commonly used to assess the nutritional status in the elderly, this study was designed to examine the mediation effect of the GNRI on the relationship between SUA level and the risk of hypertension in Chinese elderly population. It is expected that this study will provide a new perspective and evidence to prevent and screen hypertension.

## Methods

### Data source

This study used the data of wave 2009 of the China Health and Nutrition Survey (CHNS). As a national large- scale cohort study, the CHNS focused on the changes of health and nutrition, as well as the effects of the social and economic transformation on health and nutritional statuses in China. It was described in detail in the published literature elsewhere.[15].

### Study population

In the CHNS, a multistage and random cluster process was used to sample the study population from nine provinces, which were representative in geography, economic development, and health indicators. All participants were interviewed in household to collect information of lifestyle, health status, marriage and birth history, and detailed physical examinations including weight, height, arm and head circumference, and blood pressure. In the CHNS 2009, the biomarker data of 26 fasting blood measures were released including major cardiovascular biomarkers and important nutrition biomarkers, which has been published for an overview of these biomarkers.[16].

The inclusion criteria were as follows: who were aged ≥ 60 years in the time of survey; and who have complete analyzed data, including height, weight, blood pressure, and uric acid. To explore the mediation effect of malnutrition not attributed to diseases on the association of SUA level with hypertension, participants with severe wasting diseases, such as hepatic disease and severe renal insufficiency, were excluded. Overall, 2371 participants were included in the final analysis. This study was approved by the Institutional Review Board of the National Institute for Nutrition and Food Safety, China Center for Disease Control and Prevention, and University of North Carolina at Chapel Hill. Written informed consent was obtained from all subjects.

### Measurements

Physical index including height and weight were measured by the trained health staff or the members of the CHNS following standardized approach. All index were measured for three times. And the averages were used to analysis. Height and weight were employed to calculate body mass index (BMI).

A valid questionnaire was used to collect the data of health behaviors and medical history. The smoking status was checked via a question as follows: Have you ever smoked cigarettes (including hand-rolled or device-rolled)? If respondents answered “never smoked”, he or she was identified as no smoking. If respondents answered “yes”, he or she was identified as smoking. And the status of alcohol consumption was interviewed as follows: Did you drink beer or any other alcoholic beverage last year? If respondents answered “no”, he or she was identified as no alcohol consumption. If respondents answered “yes”, he or she was identified as alcohol consumption. All participants were interviewed for their education levels as follows: What is the highest level of education you have attained? According to the responses, education levels were divided into three levels as follows: primary school or none, middle school, and college or above. Physical activities in this study included martial arts, gymnastics, track and field, soccer and basketball, badminton and volleyball, and others (ping pong, Tai Chi, etc.). Each participant was asked for each activity: Do you participate in this activity? If they answered “no” for all these items, physical activity was identified as “no”. If they answered “yes” for at least one of these items, physical activity was identified as “yes”. The history of diabetes was identified via a question: Has a doctor ever told you that you suffer from diabetes? If the answer was “no”, the history of diabetes was identified as “no”. If the answer was “yes”, the history of diabetes was identified as “yes”. Ethnicity was divided into Han and others according to the participants’ identification cards.

All participants were supposed to provide the fasting blood samples in wave 2009. The automated biochemistry analyzer (Hitachi 7600, Randox, UK and Kyowa, Japan) was used to assay SUA and serum albumin (ALB) in Beijing central laboratory.

### Definitions of the GNRI and hypertension

The GNRI was calculated by ALB and weight as follows: GNRI= [ 1.489× albumin (g/L)] + [ 41.7× ( actual weight/ ideal weight)].[17] Ideal body weight was calculated by the Lorentz formula.[17] Sex-specific Lorentz formula was: ideal weight of males = height − 100 - [(height − 150)/4] and ideal weight of females = height − 100 - [(height − 150)/2.5]. If actual weight / ideal weight was more than one, actual weight / ideal weight was set to 1.

After resting in a sitting position for 5 min and selecting suitable cuff size, blood pressure was measured for three times using standardized mercury sphygmomanometers. Systolic blood pressure (SBP) corresponds with the first Korotkoff sound, and diastolic blood pressure (DBP) corresponds with the fifth Korotkoff sound. And the averages were used to define hypertension as follows: SBP/ DBP ≥ 140/ 90 mmHg or taking antihypertensive medications.[18].

### Statistical analysis

**Fig. 1:**
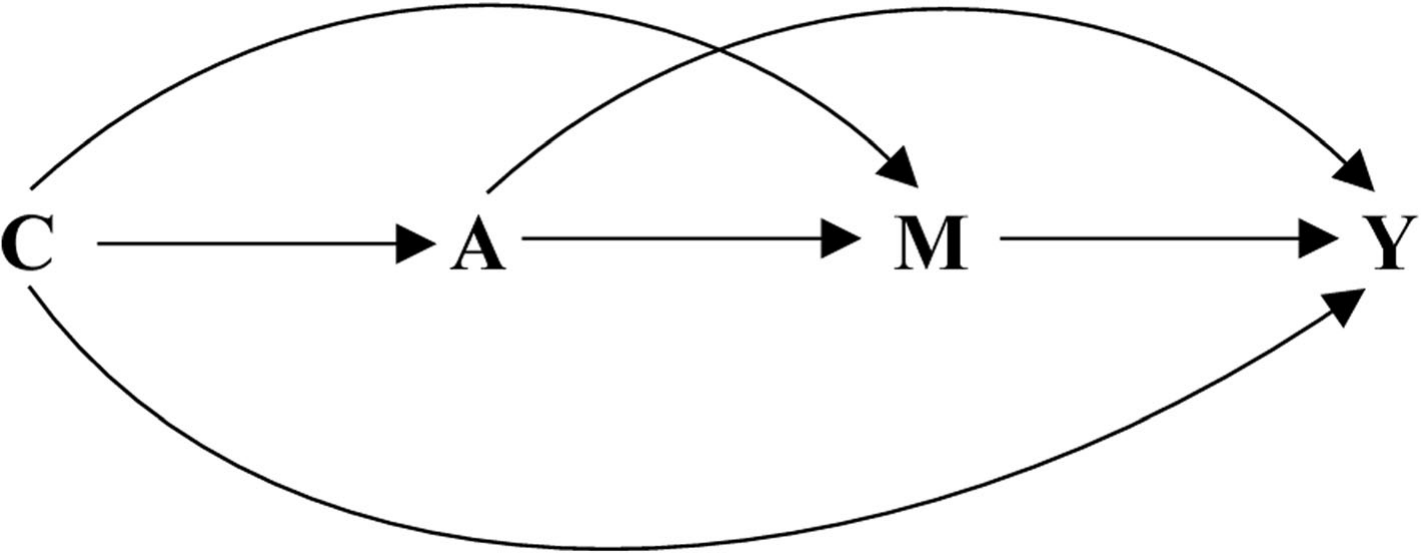
The Directed Acyclic Graph of exposure, mediation, and outcome. A = exposure; M = mediator; Y = outcome; C = covariates

Means ± standard deviations were used to describe continuous variables. And frequencies (percentages) were used to describe categorical variables. The between-group comparisons were conducted by *t-test* and *chi-square test* for continuous variables and categorical variables, respectively. Logistic regression was employed to examine the associations of the GNRI and SUA level with hypertension and obtain odd ratios (*ORs*) and 95 % confidential intervals (*CIs*). In the adjusted models, age, sex, current smoking, current drinking, education degree, physical activity, ethnicity, and history of diabetes were adjusted. In the mediate analysis, the GNRI, SUA, and hypertension were taken as the mediate variable, independent variable, and response variable, respectively. The Directed Acyclic Graph of relations among these variables is depicted in Fig. 1.

All mediation analyses were conducted using the SAS macro procedure package “*mediation analysis*”.[19] And the controlled direct effect (CDE), natural direct effect (NDE), natural indirect effect (NIE), and proportion mediated were provided. Based on the counterfactual framework, the CDE is the effect of exposure on outcome as mediator was controlled. The NDE, namely pure direct effect, is the effect of exposure on outcome with mediator was kept at the level it would naturally have taken. The NIE is the effect of mediator on outcome as exposure was controlled. When outcome is binary, the total effect is the overall effect of exposure on outcome regardless of mediator, and equals to odds ratio of the NDE multiplying by odds ratio of NIE. Similarly, the proportion mediated equals to odds ratio of the NIE divided by odds ratio of total effect. SAS 9.4 (SAS Institute Inc., Cary, NC, USA.) was employed to conduct all analyses. And a two-tailed *P* ≤ 0.05 was considered as statistical significance.

## Results

### The characteristics of all participants

A total of 2371 subjects (1120 males and 1251 females) were eligible. The mean age was 69.23 years. There were 52.64 % of participants with hypertension. The average of the GNRI was 109.16. The average of SUA level was 5.38 mg/dl. Significant differences between normal and hypertension groups were observed in all characteristics but not sex (*P* = 0.483), current drinking (*P* = 0.230), education degree (*P* = 0.965), and physical activity (*P* = 0.780) (Table 1).

**Table 1:** The characteristics of all subjects

Characteristics	All subjects (2371)	Subgroup subjects		*t/χ*^*2*^	*P*
		Non-hypertension (1123)	Hypertension (1248)		
Age (years)^※^	69.23 ± 6.77	68.30 ± 6.59	70.06 ± 6.83	-6.360	< 0.001
BMI (kg/m^2^)^※^	23.29 ± 3.68	22.44 ± 3.38	24.05 ± 3.77	-10.950	< 0.001
SUA (mg/dl)^※^	5.38 ± 1.54	5.14 ± 1.45	5.59 ± 1.60	-7.290	< 0.001
GNRI^※^	109.16 ± 6.33	108.14 ± 6.31	110.07 ± 6.20	-7.510	< 0.001
Sex^#^				0.493	0.483
Male	1120(47.24)	539 (48.00)	581 (46.55)		
Female	1251(52.76)	584 (52.00)	667 (53.45)		
Current smoking^#^				4.667	0.031
No	1760(74.26)	811 (72.22)	949 (76.10)		
Yes	610(25.74)	312 (27.78)	298 (23.90)		
Current drinking^#^				1.441	0.230
No	1752(73.89)	817 (72.75)	935 (74.92)		
Yes	619(26.11)	306(27.25)	313 (25.08)		
Education degree^#^				0.071	0.965
Primary school or none	1695(71.73)	801(71.52)	894(71.92)		
Middle school	596(25.22)	284(25.36)	312(25.10)		
College or above	72(3.05)	35(3.13)	37(2.98)		
Ethnicity^#^				12.497	< 0.001
Han	2082(87.81)	958(85.31)	1124 (90.06)		
Other	289(12.19)	165 (14.69)	124 (9.94)		
Physical activity^#^				0.078	0.780
No	2160(91.10)	1025 (91.27)	1135 (90.95)		
Yes	211(8.90)	98 (8.73)	113 (9.05)		
Diabetes history^#^				23.710	< 0.001
No	1829(77.14)	916 (81.57)	913 (73.16)		
Yes	542(22.86)	207 (18.43)	335 (26.84)		

^※^These variables were analyzed using *t test*

^#^These variables were analyzed using *chi-square test*

BMI: body mass index, GNRI: Geriatric Nutritional Risk Index, SUA: serum uric acid

### The associations of the GNRI and SUA level with hypertension

The associations of the GNRI and SUA level with hypertension are shown in Table 2. In model 1, the GNRI was associated with a higher risk of hypertension (*P* < 0.001; *OR*: 1.060; and *95 % CI*: 1.045–1.076). In model 2, there was positive association of SUA level with hypertension (*P* < 0.001; *OR*: 1.391; and *95 % CI*: 1.243–1.558). When the GNRI and SUA level were analyzed simultaneously, the result was comparable (all *P* < 0.001; *OR*: 1.055 and 1.309; and *95 % CI*: 1.039–1.071 and 1.167–1.468, respectively).

**Table 2:** The associations of the GNRI and SUA with the risk of hypertension

Factors	*β*	*P*	*OR*	*95 % CI*
**Model 1**				
GNRI	0.059	< 0.001	1.060	1.045–1.076
**Model 2**				
SUA	0.330	< 0.001	1.391	1.243–1.558
**Model 3**				
GNRI	0.054	< 0.001	1.055	1.039–1.071
SUA	0.269	< 0.001	1.309	1.167–1.468

GNRI: Geriatric Nutritional Risk Index, SUA: serum uric acid

### The mediation effect of the GNRI on the relationship between SUA level and hypertension

Table 3 shows the mediation effect of the GNRI on the relationship between SUA level and hypertension. In total population, there was a significant mediation effect (*P* < 0.001; *OR*: 1.096; and *95 % CI*: 1.048–1.146). And the proportion mediated was 17.77 %. Meanwhile, both in males and females, the results were in line with those of the total population (*P* = 0.016 and 0.001; *OR*: 1.094 and 1.093; and *95 % CI*: 1.017–1.177 and 1.035–1.155, respectively). And the proportions mediated were 18.36 % and 17.33 %, respectively.

**Table 3:** The mediation effect of the GNRI on the association of SUA with the risk of hypertension

Path		*OR*		*P*		*95 % CI*		Proportion mediated (%)	
***Whole sample (N = 2371)***									
CDE		1.799		< 0.001		1.604–2.017		-	
NDE		1.799		< 0.001		1.430–2.262		-	
NIE		1.096		< 0.001		1.048–1.146		17.77	
Total effect		1.971		< 0.001		1.562–2.488		-	
**Males** ***(N = 1120)***									
CDE		1.717		< 0.001		1.417–2.080		-	
NDE		1.717		0.006		1.170–2.519		-	
NIE		1.094		0.016		1.017–1.177		18.36	
Total effect		1.878		0.001		1.273–2.771		-	
**Females** ***(N = 1251)***									
CDE		1.802		< 0.001		1.559–2.083		-	
NDE		1.802		< 0.001		1.349–2.407		-	
NIE		1.093		0.001		1.035–1.155		17.33	
Total effect		1.970		< 0.001		1.469–2.642		-	

GNRI: Geriatric Nutritional Risk Index, CDE: controlled direct effect, NDE: natural direct effect, NIE: natural indirect effect, SUA: serum uric acid

Table 4 shows the mediation effects of the GNRI on the relationship between SUA level and hypertension stratified by age. In the 60–69 years and the 70–79 years groups, significant mediation effects of the GNRI were found (*P* = 0.002 and 0.032; *OR*: 1.099 and 1.075; and *95 % CI*: 1.036–1.165 and 1.006–1.148, respectively) but not in the 80–99 years (*P* = 0.303). And the proportions mediated were 16.22 % and 18.36 % in the 60–69 years and the 70–79 years groups, respectively.

**Table 4:** The mediation effect of the GNRI on the association of SUA with the risk of hypertension stratified by age

Path	*OR*	*P*	*95 % CI*	Proportion mediated (%)
**60–69 years** ***(N = 1403)***				
CDE	2.038	< 0.001	1.754–2.368	-
NDE	2.038	< 0.001	1.510–2.752	-
NIE	1.099	0.002	1.036–1.165	16.22
Total effect	2.239	< 0.001	1.650–3.038	-
**70–79 years** ***(N = 777)***				
CDE	1.500	< 0.001	1.227–1.833	-
NDE	1.500	0.048	1.004–2.240	-
NIE	1.075	0.032	1.006–1.148	18.36
Total effect	1.612	0.021	1.076–2.414	-
**80–99 years** ***(N = 191)***				
CDE	1.410	0.113	0.922–2.155	-
NDE	1.410	0.428	0.603–3.293	-
NIE	1.094	0.303	0.922–1.297	24.42
Total effect	1.542	0.324	0.652–3.645	-

GNRI: Geriatric Nutritional Risk Index, CDE: controlled direct effect, NDE: natural direct effect, NIE: natural indirect effect, SUA: serum uric acid

## Discussion

In this study, we found that the GNRI mediated the relationship between SUA level and hypertension. And the mediation effect was fully observed in the 60–79 years population. The proportion mediated by malnutrition was approximately 17.77 %.

It has been confirmed that elevated SUA level contributed to the development of hypertension.[20, 21] But the contribution of nutritional status was not taken into account in these studies. A recent study found that waist circumference and BMI, as obesity indicators, may mediate the relationship between SUA level and hypertension.[7]Several studies have examined the associations of abdominal obesity and visceral fat with SUA level.[22, 23] A recent study reported that a higher BMI was significantly associated with an increased risk of new-onset hyperuricemia among hypertensive patients.[24] Therefore, nutritional status might mediate the association of SUA level with hypertension through the path of BMI. Furthermore, a review declared that SUA levels were associated with adverse outcomes in the general population, including incident coronary heart disease, heart failure, atrial fibrillation, and mortality.[25] Besides cardiovascular diseases, it was documented that SUA may be a risk factor for cerebrovascular diseases including Alzheimer’s disease and Parkinson’s dementia, as well as a possible marker of malnutrition.[26] Therefore, malnutrition defined by the GNRI may modify the association of SUA level with hypertension.

On the other hand, SUA is the main final product of the common pathway of purine nucleotides metabolism,[27] and a naturally occurring antioxidant in blood.[28] An overwhelming amount of studies evidenced that the antioxidant property of ALB can inhibit production of free hydroxyl radicals and scavenge peroxy radicals.[29–31] Moreover, it has been proposed that albumin is a major known antioxidant in extracellular fluids.[32] As a result, SUA level can affect serum albumin, which is one of elements to define the GNRI. Furthermore, previous studies found that a decreased serum albumin level could predict the incidence of hypertension.[33–35] Therefore, the GNRI might also mediate the relationship between SUA level and hypertension by the path of serum albumin. Another study reported that the GNRI was associated with all-cause mortality, which can partly attribute to hypertension.[36] Therefore, there was a significant association of malnutrition with hypertension. This implied that SUA level increased the risk of hypertension partly via the GNRI. Taken together, the GNRI served as a mediator of the relationship between SUA level and hypertension by the paths of ALB and BMI.

In this study, the total effect equals to odds ratio of the NDE multiplying by odds ratio of the NIE. In the total population, odds ratio of the total effect was 1.971. Thus, the total effect of the GNRI and SUA level will result in approximately double risk of hypertension. Furthermore, odds ratio of the indirect effect of malnutrition was 1.096, which meant that the risk of hypertension will increase 9.6 % through the path of the GNRI. Generally, both of the ratio of NIE and NDE and the proportion mediated were used to assess the mediation effect. However, since the proportion mediated is suggested to be more stable than the ratio, the proportion mediated was used to assess the mediation effect of the GNRI in this study.[37] The mediation effect of the GNRI accounted for 17.77 % of the total effect. Therefore, the GNRI considerably mediated the association of SUA level with the risk of hypertension. Thus, if nutrition status was kept under the normal condition by controlling SUA level, the risk of hypertension would be reduced by 17.77 %. In this study, the potential cut-off point of SUA was 5.47 mg/dl according to the GNRI. Therefore, it implied that controlling SUA level below 5.47 mg/dl was better to prevent from hypertension.

Since this study was a cross-sectional study, it was poor evidence to conclude that the GNRI acted as a causal mediator of the relationship between SUA level and hypertension. However, a previous study reported that nutritional status modified the relationship between SUA level and CVD mortality.[38] Given hypertension is a major and independent risk factor of CVD mortality, the mediation effect of the GNRI might be explicable for the association of SUA level with hypertension.[39] The potential mechanisms might be as follows: First, the concentration of SUA was strongly associated with the protein and calorie intake, protein-energy wasting in the malnourished participants will result in a low SUA level.[38] Second, a low SUA level in the malnourished participants might be similar with deficiency of vitamins C and D, both of which are antioxidant and anti-inflammatory vitamins.[40] Therefore, a low SUA level would lead to vascular smooth muscle cell proliferation, inflammation, and oxidative stress, as well as rise blood pressure.[41] Third, since the antioxidant property and large antioxidant capacity of SUA, a low SUA level would increase blood pressure due to the decreased antioxidant capacity in the malnourished participants.[42] Taken together, we declared that the GNRI was a mediator of the association of SUA level with hypertension.

In this study, the GNRI defined by BMI and ALB was taken as a mediator but not SUA. Although the direction of the relationship between SUA level and the GNRI failed to be completely clear, there were some indirect evidences. Previous study suggested that SUA level may elevate ALB level and cause albuminuria by the paths of the activated renin–angiotensin system, endothelial dysfunction, and low-grade inflammation, which involved in the development of hypertension.[43]Furthermore, hyperuricemia can induce renal vasoconstriction and cortical ischemia to develop into preglomerular arteriolopathy and tubulointerstitial inflammation, which in turn will cause albuminuria and an increase in blood pressure.[44] On the other hand, a longitudinal study demonstrated that a high SUA level increases BMI, which was used to define the GNRI.[45] Therefore, SUA level will affect nutritional status. Given that the mentioned above, the GNRI was suitable to be a mediator of the association of SUA level with hypertension.

## Strengths and limitations

The present study had several strengths. First, due to the good representativeness of this study, the current results were comprehensive and representative of China. Second, the GNRI was defined using BMI and ALB in the present study. Thus, this indicator could fully reflect nutrition status and was more clinically significant. Therefore, the conclusion of this study was accurate and significant. Third, this was the first study to investigate the mediation effect of the GNRI on the relationship between SUA level and hypertension. Thus, this study would provide new viewpoints and evidences to prevent from hypertension.

However, there were still limitations to be noted. First, this study was based on a cross-sectional study, which was poor to examine the causal mediation effect. A longitudinal study should be further conducted to investigate the causal mediation effect of the GNRI on the association of SUA with hypertension in the future. Second, the data of dietary and history of cardiovascular diseases were unavailable in the CHNS. Therefore, the confounding effect might not be fully corrected. Third, the mechanisms of the GNRI mediating the association of SUA level with hypertension failed to be fully explained. The underlying mechanism needs to be further investigated in the laboratory. Fourth, the sample size of the 80–99 years group was not enough, which might contribute to the disappeared mediation effect of the GNRI on the relationship between SUA level and hypertension.

## Conclusions

This study provided epidemiological data to show that there was significant mediation effect of the GNRI on the relationship between SUA level and hypertension. And this mediation effect was fully observed in both males and females, especially in the 60–79 years population. Furthermore, the GNRI can account for approximately 17.77 % of the relationship between SUA level and hypertension.

## Data Availability

The datasets analyzed during the current study are available in the CHNS: http://www.cpc.unc.edu/projects/china.

## Abbreviations

SUA: Serum uric acid
GNRI: Geriatric Nutritional Risk Index
CHNS: China Health and Nutrition Survey
BMI: Body mass index
ALB: Albumin
SBP: Systolic blood pressure
DBP: Diastolic blood pressure
ORs: Odd ratios
CIs: Confidential intervals
CDE: Controlled direct effect
NDE: Natural direct effect
NIE: Natural indirect effect
