# Correction to: The geriatric nutritional risk index mediated the relationship between serum uric acid and hypertension: a mediation analysis

**DOI:** 10.1186/s12877-021-02592-1

**Published:** 2021-11-22

**Authors:** Zhongnan Cao, Sui Dai, Xun Liu

**Affiliations:** 1 Department of Cardiology, Tianjin Fifth Central Hospital, Tianjin, 300450 China; 2 Department of Laboratory, Tianjin Fifth Central Hospital, Tianjin, 300450 China; 3 Department of Ultrasonics, Tianjin Fifth Central Hospital, No 41 Zhejiang Road, Tianjin, 300450 China

**Correction to: BMC Geriatr 21, 527 (2021)**

**https://doi.org/10.1186/s12877-021-02483-5**

During the publication process of the original article [1] the following contribution statement was accidentally omitted:

- Zhongnan Cao and Sui Dai equally contributed to this work

The publisher apologizes for the inconvenience caused.
